# A lipidated peptide of *Mycobacterium tuberculosis* resuscitates the protective efficacy of BCG vaccine by evoking memory T cell immunity

**DOI:** 10.1186/s12967-017-1301-x

**Published:** 2017-10-06

**Authors:** Pradeep K. Rai, Sathi Babu Chodisetti, Weiguang Zeng, Sajid Nadeem, Sudeep K. Maurya, Susanta Pahari, Ashok K. Janmeja, David C. Jackson, Javed N. Agrewala

**Affiliations:** 1 CSIR-Institute of Microbial Technology, Chandigarh, India; 2 Present Address: Department of Microbiology and Immunology, Pennsylvania State University College of Medicine, Hershey, PA 17033 USA; 3 Department of Microbiology and Immunology, Peter Doherty Institute for Infection and Immunity, The University of Melbourne, Parkville, VIC 3010 Australia; 4 Department of Pulmonary Medicine, Government Medical College and Hospital, Chandigarh, India

**Keywords:** Vaccine, BCG, L91, TB, Mtb, Th1, Th17, Memory T cells

## Abstract

**Background:**

The current BCG vaccine induces only short-term protection against *Mycobacterium tuberculosis* (*Mtb*), suggesting its failure to generate long-lasting memory T cells. Previously, we have demonstrated that a self-adjuvanting peptide of *Mtb* (L91), successfully generated enduring memory Th1 cells. Consequently, we investigated if L91 was able to recuperate BCG potency in perpetuating the generation of memory T cells and protection against *Mtb* infected mice.

**Methods:**

In the present study, we evaluated the potency of a self adjuvanting *Mtb* peptide vaccine L91 in invigorating BCG immune response against *Mtb* in mice. Female BALB/c mice were immunized with BCG. Later, they were boosted twice with L91 or an antigenically irrelevant lipidated influenza virus hemagglutinin peptide (LH). Further, PBMCs obtained from BCG vaccinated healthy subjects were cultured in vitro with L91. T cell responses were determined by surface markers and intracellular cytokine staining. Secretion of cytokines was estimated in the culture supernatants (SNs) by ELISA.

**Results:**

Compared to the BCG-vaccinated controls, L91 booster significantly enhanced the percentage of memory Th1 cells and Th17 cells and reduced the mycobacterial burden in BCG primed and L91-boosted (BCG-L91) group, even after 229 days of BCG vaccination. Further, substantial augmentation in the central (CD44^hi^CD62L^hi^CD127^hi^) and effector memory (CD44^hi^CD62L^lo^CD127^lo^) CD4 T cells was detected. Furthermore, greater frequency of polyfunctional Th1 cells (IFN-γ^+^TNF-α^+^) and Th17 cells (IFN-γ^+^IL-17A^+^) was observed. Importantly, BCG-L91 successfully prevented CD4 T cells from exhaustion by decreasing the expression of PD-1 and Tim-3. Additionally, augmentation in the frequency of Th1 cells, Th17 cells and memory CD4 T cells was observed in the PBMCs of the BCG-vaccinated healthy individuals following in vitro stimulation with L91.

**Conclusions:**

Our study demonstrated that L91 robustly reinvigorate BCG potency to invoke enduring protection against *Mtb*. This novel vaccination stratagem involving BCG-priming followed by L91-boosting can be a future prophylactic measure to control TB.

**Electronic supplementary material:**

The online version of this article (doi:10.1186/s12967-017-1301-x) contains supplementary material, which is available to authorized users.

## Background

Tuberculosis (TB) is a deadly disease infecting 9.6 million people globally and accounts for 1.5 million deaths annually [1]. Currently, BCG is the only available vaccine against TB, which is administered since 1974 under immunization program of World Health Organization (WHO) [2, 3]. Unfortunately, continuous increase in the number of TB cases raises a question on the protective efficacy of BCG [4–6]. Interestingly, BCG protects children from TB [7], indicating that it has adequate antigenic repertoire to protect against *Mycobacterium tuberculosis * (*Mtb*). In contrast, it fails to safeguard adults from TB; which has been suggested to be due to its failure to generate long-lasting memory T cells [8].

Many studies are in progress to bolster BCG efficiency to impart enduring immunity. Memory inducing cytokines like IL-7 and IL-15 have been shown to sufficiently augment the BCG induced memory T cells [8]. Furthermore, booster dose of *Mtb* antigen Acr1 entrapped in fusogenic-liposomes generated long-term memory T cells and improved BCG potency [9]. Thus, it implies that the protective efficacy of BCG can be boosted through antigen-priming. Recently, we have synthesized a novel lipopeptide vaccine construct L91, which comprises of a promiscuous-peptide derived from Acr1 and the TLR2 agonist Pam2Cys [5, 10]. L91 elicited both innate and adaptive immunity successfully through its Pam2Cys and peptide component, respectively [5, 10]. TLR-2 promotes the generation of memory T cells, rescued Th1 cells from exhaustion and protected mice from chronic TB [11]. Intriguingly, L91 elicited long-lasting memory T cells and protected mice and Guinea pigs from *Mtb* infection [10].

In the current study, we have demonstrated that the memory T cell generation and protection efficacy of BCG vaccine against *Mtb* could be significantly bolstered with L91 boosting of the BCG-vaccinated population. Specifically we observed improvement in the pool of enduring memory Th1 and Th17 responses, the cells that play crucial role in protection against *Mtb*. In future, this vaccination strategy may be implemented to protect people from TB.

## Methods

### Study design

Female BALB/c mice (6–8 week) were procured from the Experimental Animal Facility, CSIR-Institute of Microbial Technology, Chandigarh, India. Mice were immunized subcutaneously (sc) at the base of tail with the Danish strain of BCG (10^6^ CFU/mouse). Twenty-one days later, BCG-primed mice were boosted twice with L91, at an interval of 14 days apart (BCG-L91). Control groups were immunized with BCG alone (BCG), placebo (PBS) or an antigenically irrelevant lipidated influenza virus hemagglutinin peptide (abbreviated as LH or Pam2Cys). Mice were aerosol challenged with *Mtb* (~100 CFU/mouse), 90 days after the last booster. Subsequently, animals were sacrificed after 90 days of *Mtb* challenge. Later, immunological (ex vivo), protection and histopathology studies were performed. To monitor the antigen specific T cell response, mice were sacrificed 30 days after *Mtb* infection, and cellular responses were examined following in vitro stimulation with L91, Pam2Cys and short term culture filtrate of H37Rv (ST-CF). In all the experiments, changes in the response on vaccination were compared among BCG-L91 and control BCG and placebo (PBS) groups or otherwise indicated.

### Vaccine constructs used in study

Lipidated synthetic peptides used in the study were produced by solid phase synthesis method, as described elsewhere [12]. The lipidated promiscuous peptide of sequence SEFAYGSFVRTVSLPVGADE was from the Acr1 antigen of *Mtb* (L91). The control, non-mycobacterial, lipidated peptide (LH) sequence ALNNRFQIKGVELKS was from influenza virus hemagglutinin light chain and was shown to be active in BALB/c mice [13].

### Mycobacterial strains and BCG

*Mtb* H37Rv strain was cultured in 7H9 medium containing Tween-80 (0.05%), supplemented with albumin (10%), dextrose and catalase (ADC). Glycerol stocks of H37Rv were prepared and stored at −80 °C, and later used for infection studies. BCG vaccine (TUBERVAC) used for immunization was purchased from Serum Institute of India, Pune, India. TUBERVAC (*Bacillus Calmette*-*Guerin* Vaccine I.P.) is a live freeze-dried vaccine derived from an attenuated strain of *Mycobacterium bovis* and meets the requirements of WHO and I.P. when tested by the methods outlined in WHO, TRS. 745 (1987), 771 (1988) and I.P.

### Reagents and antibodies

Chemicals and reagents were purchased from Sigma-Aldrich (St. Louis, MO). Anti-mouse or anti-human fluorochrome labeled antibodies (Abs): CD4-PB, CD62L-APC, CD44-PerCP-Cy5.5, CD127-PE, FoxP3-FITC, Tim3-PE, PD1-PECy7, IFN-γ-PECy7, TNFα-PerCPCy5.5, IL-17-PerCPCy5.5, CD25APC-Cy7, CD45RA-PE, CD45RO-APC, and Abs for ELISA were procured from BD Pharmingen (San Diego, CA) or otherwise mentioned. RPMI-1640 and FBS were purchased from GIBCO (Grand Island, NY) for cell culture. For culturing of cells, tissue culture grade plastic-wares were purchased from BD Biosciences (Bedford, MA). Ab against iNOS used in Western blot was procured from (Abcam, Cambridge, United Kingdom).

### Isolation of lymphocytes from lymph nodes, spleen and lungs

Spleens and LNs obtained from the immunized mice and exposed to *Mtb*, were pooled and single cell suspension was prepared by gently pressing through frosted slides. Lungs were perfused with chilled PBS and small pieces were prepared and digested with collagenase (2 mg/ml) for 30 min/37 °C. Later, cells were passed through a sieve (70 μM). Viability was checked by trypan blue dye-exclusion method and cells (2 × 10^5^/well) were added to 96 well U-bottom culture plates and cultured with L91 (1 nmol), Pam2Cys (50 ng/ml), ST-CF (25 μg/ml) and medium for 72 h.

### Proliferation assays

The cells (2 × 10^7^ cells) were incubated with carboxyfluoresceinsuccinimidyl ester (CFSE) dye in PBS (1 μM, 4 ml) at 37 °C. Free CFSE was quenched with 2 ml of FCS and excess was removed by washing with RPMI-FCS-10%. CFSE-labeled cells were cultured with either L91 (1 nmol), Pam2Cys (50 ng/ml), ST-CF (25 μg/ml) or medium for 72 h. The proliferation of CFSE-labeled cells was analyzed by flow cytometry.

### Intracellular cytokine and surface staining

The cells were stimulated as mentioned in the proliferation assay and then stimulated with PMA (50 ng/ml) and ionomycin (10 μM) for 4 h followed by incubation with brefeldin A (5 mg/ml) for an additional 2 h. The cells were then harvested, washed twice with buffer (PBS-FCS-2%) and fixed with paraformaldehyde (1X) at 4 °C for 30 min. Fixed cells were perforated with saponin (0.2%) and incubated with fluorochrome tagged anti-IFN-γ, IL-17A and TNF-α Abs at 4 °C for 90 min. The cells were washed with saponin (0.2%), followed by wash buffer. For surface staining, the cells were incubated with either fluorochrome labeled Abs or biotinylated Abs/streptavidin-fluorochrome conjugates. Standard protocols of washing/incubation were followed at each stage.

### Flow cytometry

Flow cytometry was carried out using a FACS-Aria III and data was analyzed using the BD FACS DIVA software package (BD Biosciences, San Jose, CA). The gating strategies for FoxP3, PD-1, Tim-3, IFN-γ/TNF-α, IFN-γ/IL-17, CD62L/CD44, CD127 (Additional file 1: Figure S1) and CCR6/CXCR3 expression on IL-17/IFN-γ double positive cells (Additional file 2: Figure S2) have been shown in their respective figures.

### Cytokine estimation

The cultures were set as mentioned in T cell proliferation assay. Later, culture supernatants (SNs) were collected and cytokine concentrations were determined using a standard sandwich ELISA [14].

### Culture of dendritic cells

Monocytes were isolated from the femurs and tibia of the mice. The cells (2 × 10^6^/well) were cultured in the presence of granulocyte macrophage colony-stimulating factor (GM-CSF; 2 ng/ml) and interleukin-4 (IL-4; 4 ng/ml) for the generation of DCs. On day 3, the cultures were replenished with fresh medium and supplemented with GM-CSF and IL-4. On day 6, the cells were harvested, washed and added to 24 well plates (2 × 10^5^ cells/well). Bone marrow derived dendritic cells (BMDCs) were stimulated with either L91 (3 nmol), F91 (3 nmol), Pam2Cys (50 ng/ml) or LPS (4 μg/ml) for 16 h. SNs were collected for cytokines estimation by ELISA and the expression of surface markers was assessed by flow cytometry.

### Western blotting for iNOS

As mentioned above for the cultures of DCs and macrophages, BMDCs were stimulated with L91, F91, Pam2Cys and LPS for 16 h in 12 well plate (10^6^ cells/well). The cells were harvested and lysed in radioimmunoprecipitation assay buffer (RIPA) containing a protease inhibitor cocktail. Protein was estimated and equal amounts (30 μg) were subjected to SDS-PAGE (10%) followed by transfer to PVDF membrane. The non-specific sites were blocked with BSA (5%) and the blot was probed with anti-iNOS Abs (1:200) (Ab3523) or actin as a control. Later, the blot was probed with anti-rabbit-HRP Ab and finally developed using an enhanced chemiluminescence method (Lumigen, Inc. Southfield, MI). The blot was finally scanned using Image Quant LAS 4000 (GE Healthcare, Pittsburgh, PA).

### Demonstration of NF-κB by EMSA

BMDCs were stimulated with L91 (9 nmol), F91 (9 nmol), Pam2Cys (150 ng/ml) or LPS (4 μg/ml) for 30 min in 12 well plates (10^6^ cells/well). The cells were harvested and nuclear extract was prepared. Nuclear extracts of each sample were incubated with [P^32^] labeled oligonucleotides containing the binding site for NF-κB at 37 °C for 20 min to allow the formation of DNA–protein complexes, which was then resolved by native gel electrophoresis using a 6% gel. Later, the gel was dried and exposed to a blank screen at room temperature for 6–10 h and scanned by a phosphorimager (Fujifilm, Tokyo, Japan).

### Isolation and culture of human PBMCs

Blood was obtained from BCG vaccinated healthy volunteers in sterile vacutainers. Blood was diluted with PBS in 1:1 ratio and overlaid on histopaque. Peripheral blood mononuclear cells (PBMCs) were separated by centrifugation at 400 g for 30 min at 25 °C. PBMCs were washed 3 times with PBS + 2% FCS and in vitro cultured with L91 (1 nmol), F91 (1 nmol) or Pam2Cys (50 ng/ml) for 96 h. During culture, IL-2 (100U) was added after 24 h.

### Statistical analysis

The data are presented as mean ± standard error. Statistical analysis was performed using Graph Pad Prism employing an unpaired Student’s ‘t’test.

## Results

### L91 administration limits the generation of regulatory T cells

**Fig. 1:**
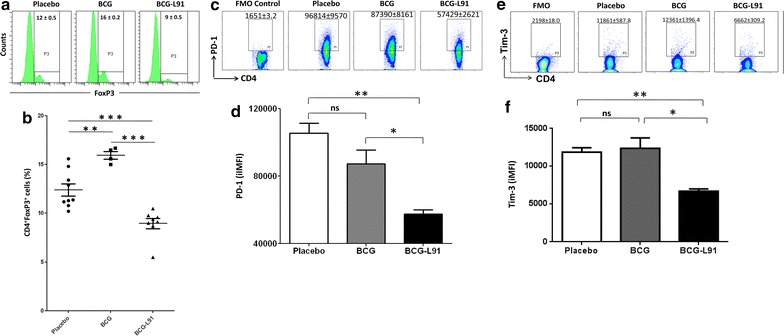
BCG-L91 limits the generation of Tregs and rescues CD4 T cells from exhaustion. The mice primed and boosted with BCG-L91 and infected with *Mtb* were sacrificed. The control animals were immunized with either BCG or placebo. A single cell suspension was prepared from lungs and ex vivo examined for the expression of **a** FoxP3; **c** PD-1; **e** Tim-3 by flow cytometry. **b** Scatter dot plot depicts percent population of FoxP3^+^ CD4 T cells. The figures (Mean ± SE) in the inset the percentage of positive cells. Each dot in the scatter plot signifies one mouse. The bar diagrams correspond to the iMFI for **d** PD-1; **f** Tim-3. Data are pooled from 2 independent experiments and shown as Mean ± SEM. *p ≤ 0.05, **p ≤ 0.005, ***p ≤ 0.0001, *ns* not significant

One of the main reasons associated with BCG failure is the generation of regulatory T cells (Tregs) following vaccination, which hampers its protective efficacy [5]. Consequently, we examined ex vivo generation of regulatory T cells (Tregs) in the cells isolated from the lungs of the immunized mice after 90 days of *Mtb* infection. We observed significantly (*p* ≤ 0.005) higher percentage of FoxP3^+^ CD4 Tregs from the BCG vaccinated mice, as compared to the control (placebo) group (Fig. 1a, b). On the other hand, a significant reduction of the FoxP3^+^ CD4 Tregs (*p* ≤ 0.0001) was observed in the group of mice that was primed with BCG followed boosting with L91 (BCG-L91), as compared to BCG. Thus, indicating that L91 boosting restricted BCG-mediated induction of Tregs and thereby may be augmenting immunity against *Mtb*.

### L91 rescues CD4 T cells from exhaustion

*Mycobacterium tuberculosis* is known to induce exhaustion of T cells [11]. Recently, we have demonstrated that signaling via TLR-2 rescues CD4 T cells from exhaustion [11]. L91 comprises of TLR-2 agonist Pam2Cys and a MHC-II binding promiscuous peptide of Acr1 [10]. Therefore, we monitored whether BCG-L91 vaccination could rescue CD4 T cells from exhaustion against *Mtb* infection. The results showed a substantial reduction of the exhaustion markers PD-1 (*p* ≤ 0.05) (Fig. 1c, d) and Tim-3 (*p* ≤ 0.05) (Fig. 1e, f) on the CD4 gated T cells obtained from BCG-L91 group, compared to those of BCG group (Fig. 1c–f). There was no difference in the expression of these two markers on the cells isolated from BCG and the placebo group. These results demonstrate that L91-boosting prevented T cell exhaustion in *Mtb* infected mice.

### BCG-L91 elicits predominantly Th1 and Th17 immunity

**Fig. 2:**
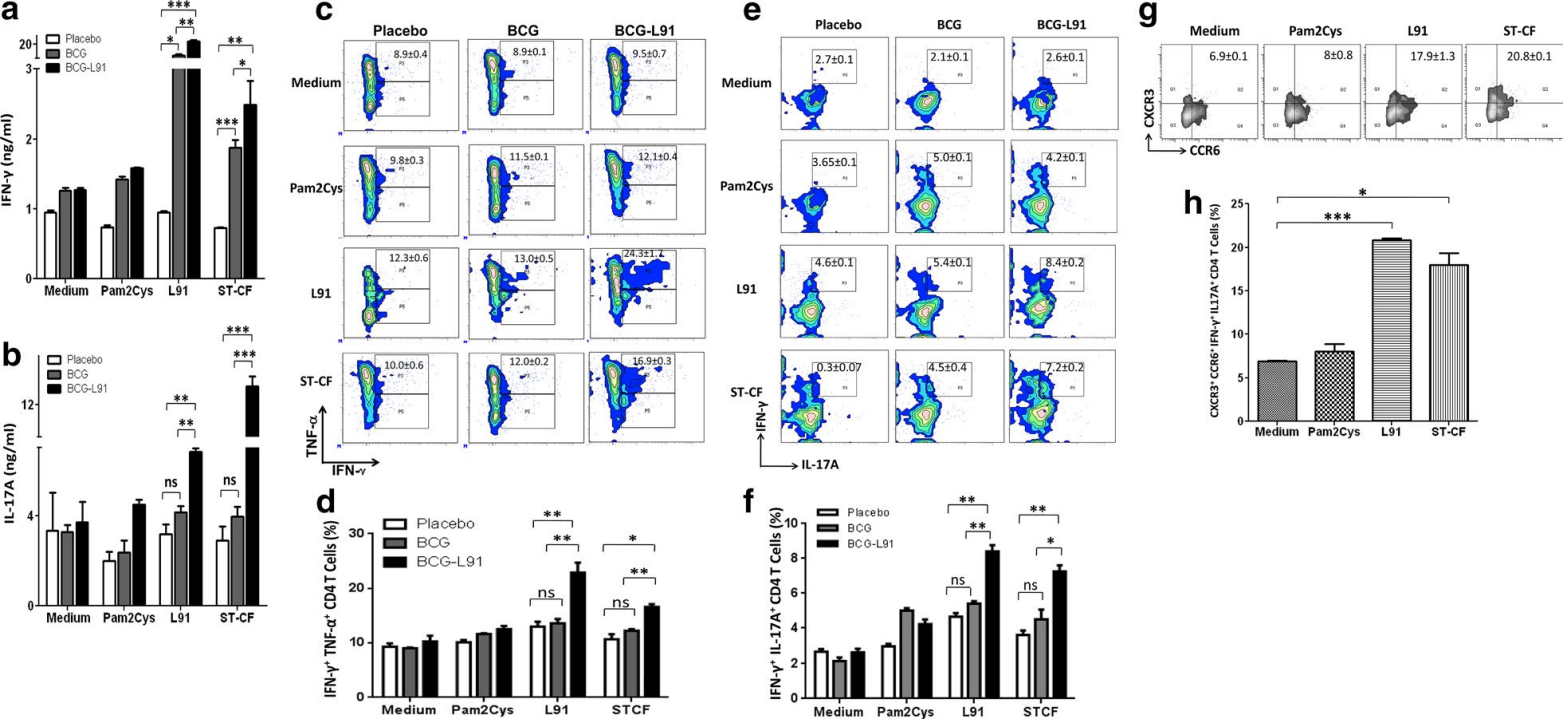
BCG-L91 generates polyfunctional Th1 cells and Th17 cells. The mice primed and boosted with BCG-L91 and infected with *Mtb* were sacrificed. The control animals were only immunized with either BCG or placebo. A single cell suspension was prepared from lungs and in vitro incubated with L91, Pam2Cys and ST-CF for 72 h. Secretion of IFN-γ and IL-17A was examined in the culture SNs by ELISA. The bar diagram indicates amount of **a** IFN-γ and **b** IL-17A secreted by lungs cells. The co-expression of **c** IFN-γ + TNF-α; **e** IL-17A + IFN-γ was checked by flow cytometry on CD4 gated T cells. Bar diagrams represent the percent of double positive cells expressing **d** IFN-γ + TNF-α; **f** IL-17A + IFN-γ on CD4 gated T cells of lungs. **g** The flow cytometry contours and **h** bar diagram designates the co-expression of CXCR3 + CCR6 on IFN-γ + IL-17A polyfunctional CD4 T cells. Data are representative of 3 independent experiments and shown as Mean ± SEM. *p ≤ 0.05, **p ≤ 0.005, ***p ≤ 0.0005, *ns* not significant

Th1 cells and Th17 cells are two major arms of adaptive immunity that play a cardinal role in safeguarding against *Mtb* [15]. Th1 cells are known to mediate protection against *Mtb* through the release of IFN-γ and TNF-α [7, 10, 16]. Th17 cells confer protection by chemokine mediated recruitment of the cells of innate and adaptive immunity [17, 18]. BCG has shown to elicit a mixed Th1 and Th2 response and also a weak Th17 response [19, 20]. Our results show that L91 booster significantly augmented Th1 and Th17 immunity, as evidenced by a greater release of IFN-γ following in vitro stimulation with L91 (*p* ≤ 0.005) or ST-CF (*p* ≤ 0.05) and IL-17A (in vitro stimulation with L91: *p* ≤ 0.005; ST-CF: *p* ≤ 0.0005) by lung cells (Fig. 2a, b) than the control groups. Thus, BCG-primed and antigen-boost strategy generated better immunity than BCG alone against *Mtb* [17, 21].

### Induction of multifunctional Th1 cells and Th17 cells by BCG-L91

Polyfunctional Th1 cells and Th17 cells are considered better in protecting against *Mtb,* compared to their counter parts secreting single cytokine [22, 23]. In our study here, the cells isolated from the lungs of the BCG-L91 group showed significantly greater expansion in the percentage of multifunctional Th1 cells (IFN-γ^+^TNF-α^+^) (L91: *p* ≤ 0.005, ST-CF: *p* ≤ 0.005) and Th17 cells (IL-17^+^IFN-γ^+^) (L91: *p* ≤ 0.005, ST-CF: *p* ≤ 0.05) following in vitro stimulation with either L91 or ST-CF, compared to the BCG group (Fig. 2c–f).

### Multifunctional Th17 cells express CCR6^hi^CXCR3^hi^ phenotype

Multifunctional Th17 cells (IFN-γ^+^IL-17A^+^) co-expressing CXCR3/CCR6 are known to upregulate the ligands for CXCR3 (CXCL9/CXCL10/CXCL11) on the lung parenchymal cells, which helps to recruit Th1 cells to the site of infection [17, 18]. The results of our study showed that significantly more CXCR3^+^CCR6^+^ co-expressing polyfunctional Th17 cells (IFN-γ^+^IL-17A^+^) had been observed in the lungs of the BCG-L91 group following culturing with L91 (*p* ≤ 0.0005) and ST-CF (*p* ≤ 0.05) (Fig. 2g, h).

### BCG-L91 enhances the frequency of T cell memory response

**Fig. 3:**
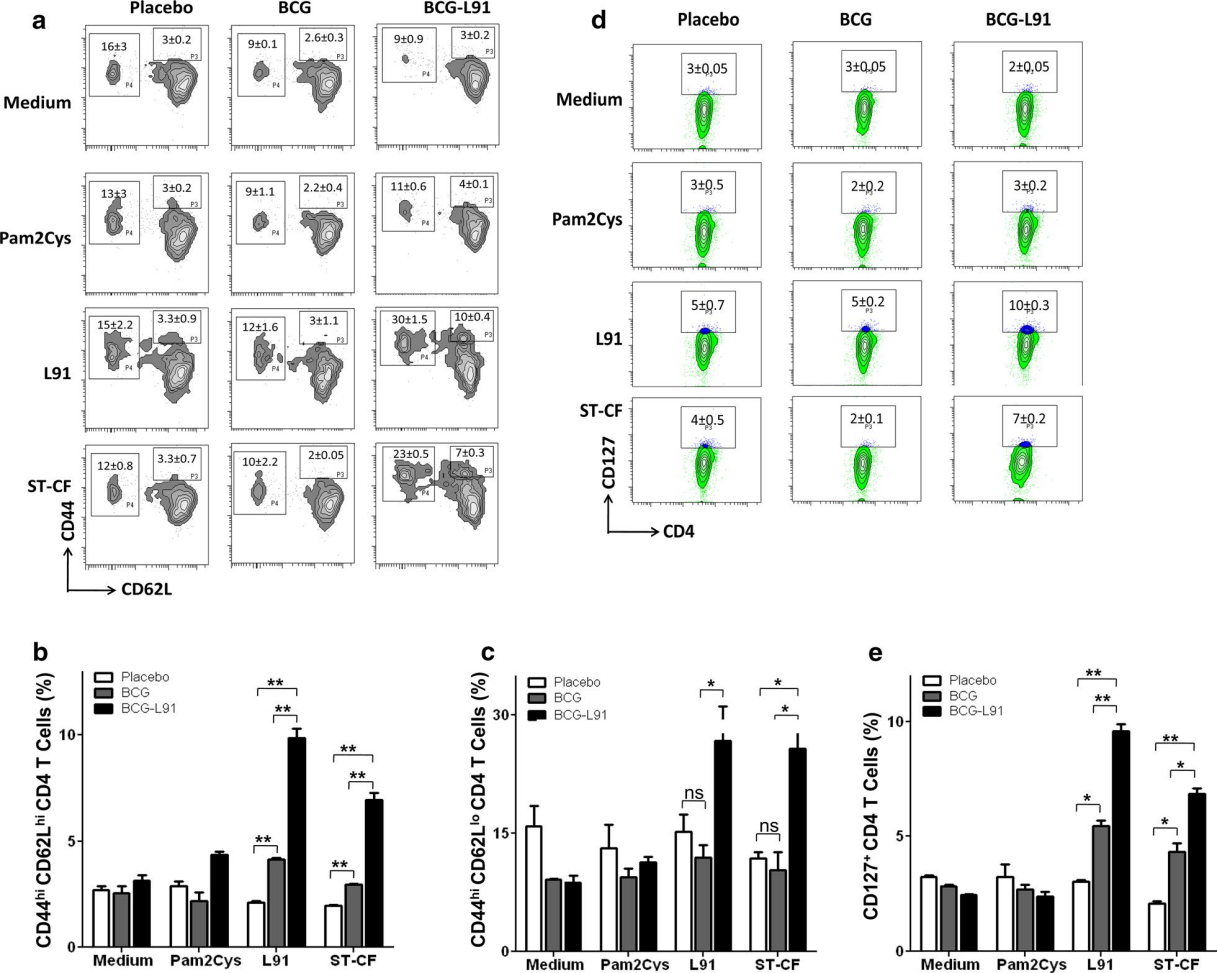
BCG-L91 elicits long lasting memory generation. The mice primed and boosted with BCG-L91 and infected with *Mtb* were sacrificed. The control animals were immunized with either BCG or placebo. A single cell suspension was prepared from lungs and in vitro incubated with L91, Pam2Cys and ST-CF for 72 h. The gated population of CD4 T cells was checked for the expression of memory markers CD62L, CD44 and CD127 by flow cytometry. **a** The flow cytometry contours and **b** bar diagram represents percent population of central memory (CD44^hi^CD62L^hi^) and **c** effector memory (CD44^hi^CD62L^lo^) CD4 T cells of the spleen. **d** CD4 gated T cells were examined for the expression of CD127 by flow cytometer on spleen cells and **e** the bar diagram depicts the percentage population of CD127 expressing CD4 T cells. Data shown in the inset are Mean ± SEM and representative of 3 independent experiments. BCG-L91: mice primed with BCG and boosted with L91. *p ≤ 0.05, **p ≤ 0.005, *ns* not significant

One of the main reasons for BCG’s inability to evoke long-term protection against *Mtb* is its failure to generate enduring memory T cells [5, 10]. The results of this study show that L91 induced significantly greater expansion in the pool of both central (CD44^hi^CD62L^hi^; L91: *p* ≤ 0.05; ST-CF: *p* ≤ 0.005) and effector memory (CD44^hi^CD62L^lo^; L91: *p* ≤ 0.05; ST-CF: *p* ≤ 0.05) CD4 T cells in the BCG-L91 administered mice than the control group following in vivo stimulation of L91 and ST-CF (Fig. 3a–c). These results were further substantiated by significantly (*p* ≤ 0.005) higher display of CD127, another marker for memory CD4 T cells (Fig. 3d, e). No discernible change was detected in the case of BCG group. The CD127 has been established to be an important marker for the sustenance of memory CD4 T cells [24].

### L91 reduces the suppression of antigen presenting cells by enhancing NF-κB and iNOS expression

**Fig. 4:**
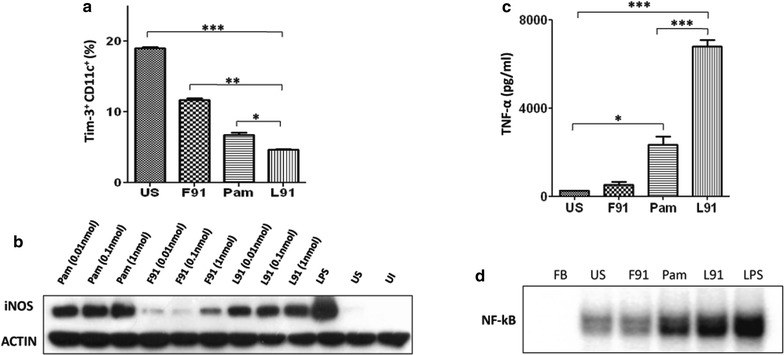
L91 suppresses Tim-3 and induces iNOS and NF-κB on DCs. Bone marrow derived DCs were stimulated with L91, F91, Pam2Cys and LPS. The expression of Tim-3 on CD11c gated population was checked by flow cytometry. **a** Bar diagram indicates percent of Tim-3^+^ population on CD11c^+^ DCs. **b** The expression of iNOS was monitored by Western blotting. **c** The secretion of TNF-α was measured in the culture SNs of DCs by ELISA. **d** The expression of NF-κB was examined by EMSA. *FB* free probe, *US* unstimulated cells, *Pam* Pam2Cys, *F91* non-lipidated peptide, *L91* lipidated peptide, *F91* non-lipidated, *Pam* Pam2Cys, *LPS* lipopolysaccharides. Data shown are representative of 2 comparable experiments and correspond to Mean ± SEM. *p ≤ 0.05, **p ≤ 0.005, ***p ≤ 0.0005, *ns* not significant

The Acr1 antigen of *Mtb* is known to tolerize dendritic cells by upregulating the inhibitory molecule Tim-3 and consequently suppresses the host immunity [25]. L91 is derived from Acr1. Hence, it was worth to examine the influence of L91 on Tim-3 expression on dendritic cells. Our results show that in vitro stimulation with L91 significantly (*p* ≤ 0.0005) downregulated the expression of Tim-3 compared to unstimulated cultures (Fig. 4a). L91 comprises of the components that are capable of inducing both innate and adaptive immunity [10]. Innate immunity is the first line of defense to combat against any pathogen [26]. Nitric oxide (NO) mediated killing of *Mtb* is one of the well-established protection mechanism [27]. Hence, we thought it would be worth to check the impact of L91 on DCs in inducing the release of NO. Interestingly, we observed a dose-dependent increase in iNOS, the enzyme responsible for NO synthesis (Fig. 4b) upon stimulation with L91. Additionally, L91 considerably (*p* ≤ 0.0005) augmented TNF-α secretion (Fig. 4c). TNF-α is a major factor in restricting the intracellular survival of *Mtb* [7, 25]. Further, the augmentation in the expression of NF-κB was also detected with L91 stimulation (Fig. 4d). NF-κB is a key transcription factor responsible for the propagation of cells and pro-inflammatory responses [28]. Besides L91, considerably higher NF-κB expression was detected following stimulation with the Pam2Cys control but not un-lipidated peptide (F91). These results together demonstrate the role of Pam2Cys as a component of L91 in promoting the release of molecules of innate immunity responsible for protection.

### L91 boosting enhances protective efficacy of BCG vaccine

**Fig. 5:**
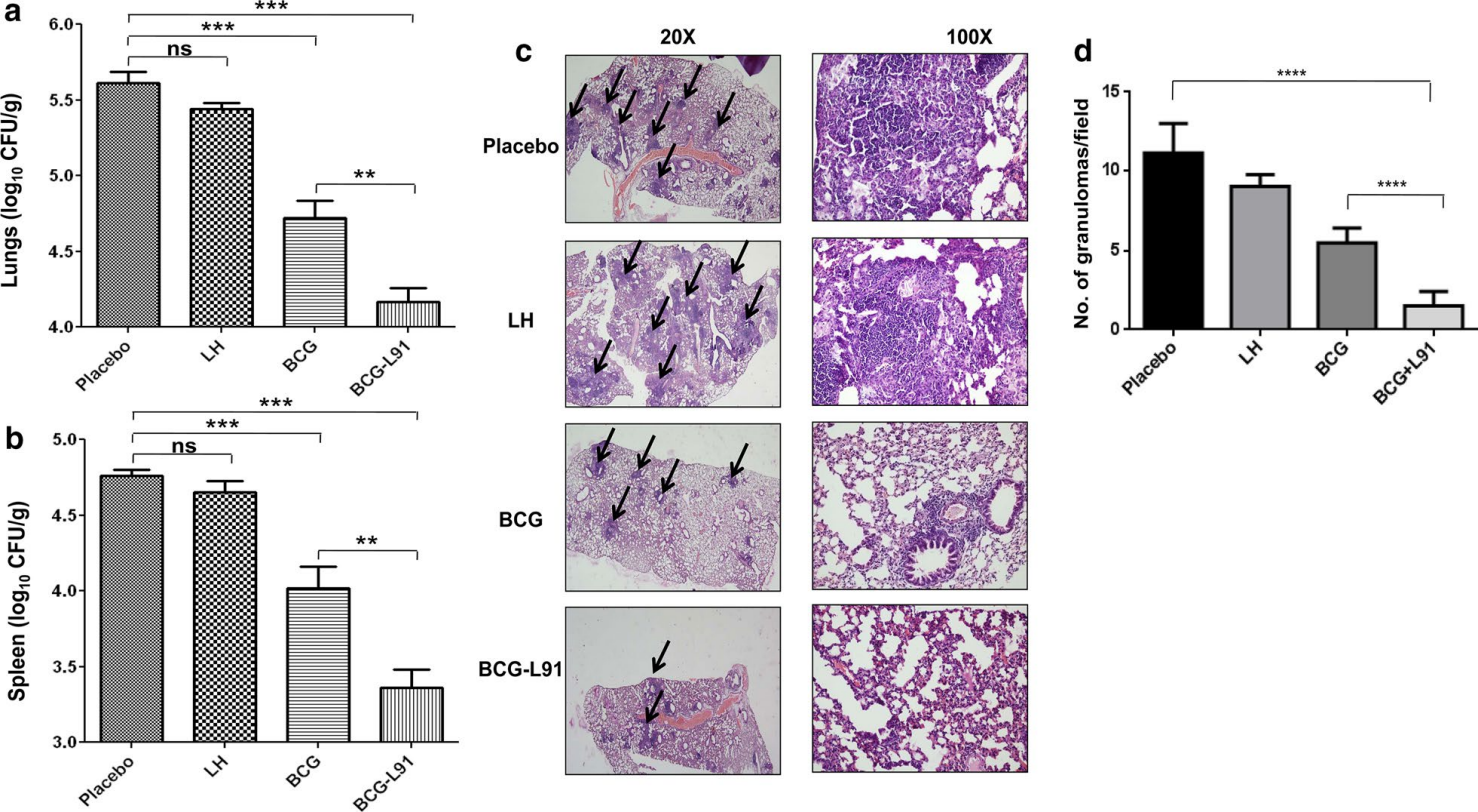
Prime boosting with BCG-L91 improves the protective efficacy of BCG. Animals were vaccinated with BCG and later boosted twice with L91. Ninety days of last booster, the mice were aerosol challenged with *Mtb*. After 90 days, bacterial burden was enumerated in the **a** lungs and **b** spleen. Control animals received saline, LH and BCG. The bacterial number is expressed as log_10_CFU/g of tissue. Results shown as bar diagram are Mean ± SEM and pooled data from 3 experiments (n = 3 mice/group). **c** Histopathological analysis of lung was done after staining the sections with hematoxylin and eosin. The magnification is depicted at 20 ×/100 × for lungs. The arrows indicate granulomas or tubercle. The asterisk represents the level of significance. **d** The quantitative assessment of the *Mtb* infected lungs was done by counting the granulomas in ten random fields of the gross histopathological sections and the average is expressed as Mean ± SD. *LH* lipidated hemagglutinin peptide, *BCG-L91* mice primed with BCG and boosted with L91. **p ≤ 0.005, ***p ≤ 0.0005, *ns* not significant

After establishing the role of L91 in inducing the long-lasting memory T cell response, we next evaluated the protective efficacy of BCG-L91 against *Mtb*. To generate bona fide memory T cell response, BCG-L91 vaccinated mice were aerosol challenged with *Mtb* 90 days after the last immunization. Bacterial burden in the lungs and spleen was enumerated 90 days following the challenge. The results show that L91 boosting led to a greater reduction in the mycobacterial burden in the lungs (*p* ≤ 0.005) and spleen (*p* ≤ 0.005), compared to BCG-vaccinated group, and also the Pam2Cys and placebo control (Fig. 5a, b). The decrease in the CFU in the spleen also signifies the importance of BCG-L91 in restricting the dissemination of *Mtb*.

Further, we substantiated CFU data by histopathological study of lungs. Animals immunized with BCG-L91 exhibited reduced pathology in the lungs, manifested by decreased macrophage infiltration and lesser and smaller size of granulomas, fewer scattered peribronchiolar lymphocyte-rich areas and better preserved alveolar spaces, when compared to the BCG group, placebo and Pam2Cys controls. The control animals revealed *Mtb* induced severe pathology that was characterized by the larger size and number of lymphocyte rich compact granulomas with signs of irregular lung architecture due to excessive inflammation. Peribronchiolar cuffs and bigger confluent areas of consolidated mixed lymphocytes-histiocytes were also apparent (Fig. 5c). The quantitative assessment of the granulomas in lung sections revealed a decreased *Mtb* burden in BCG-L91 immunized animals (Fig. 5d).

### L91 augments the expansion and activation of human CD4 T cells

**Fig. 6:**
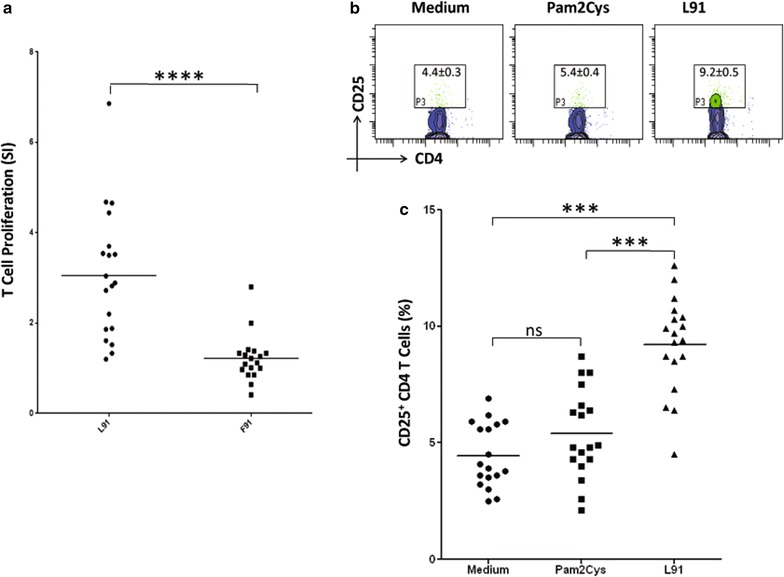
L91 induces proliferation, activation and generation of memory CD4 T cells of BCG vaccinated volunteers. PBMCs from healthy BCG immunized individuals were stimulated with L91 and control cultures with F91, Pam2Cys or media alone for 96 h and cells were analyzed for **a** proliferation by ^3^H-thymidine incorporation (n = 20); **b** contour plots and **c** scatter dot plot shows percentage population of CD25 expressing CD4 T cells on in vitro stimulation with L91 (n = 18). The data in the inset of dot plots illustrate Mean ± SEM of CD25^+^ CD4 positive cells. Each dot in scatter plots corresponds to one human subject. Data are shown as Mean ± SEM. ***p ≤ 0.0005, ***p ≤ 0.0005, *ns* not significant

L91 efficiently amplified BCG induced immunity in the experimental model of TB. Hence, we were next curious to know whether L91 was able to activate human CD4 T cells obtained from BCG vaccinated healthy volunteers. PBMCs cultured with L91 exhibited significantly higher (*p* ≤ 0.0005) proliferation and upregulation of the activation marker CD25 (*p* ≤ 0.0005) on CD4 T cells, as compared to those cultured with free peptide or Pam2Cys control (Fig. 6a–c).

### L91 enhances the generation of enduring polyfunctional Th1 cells and Th17 cells in human PBMC

**Fig. 7:**
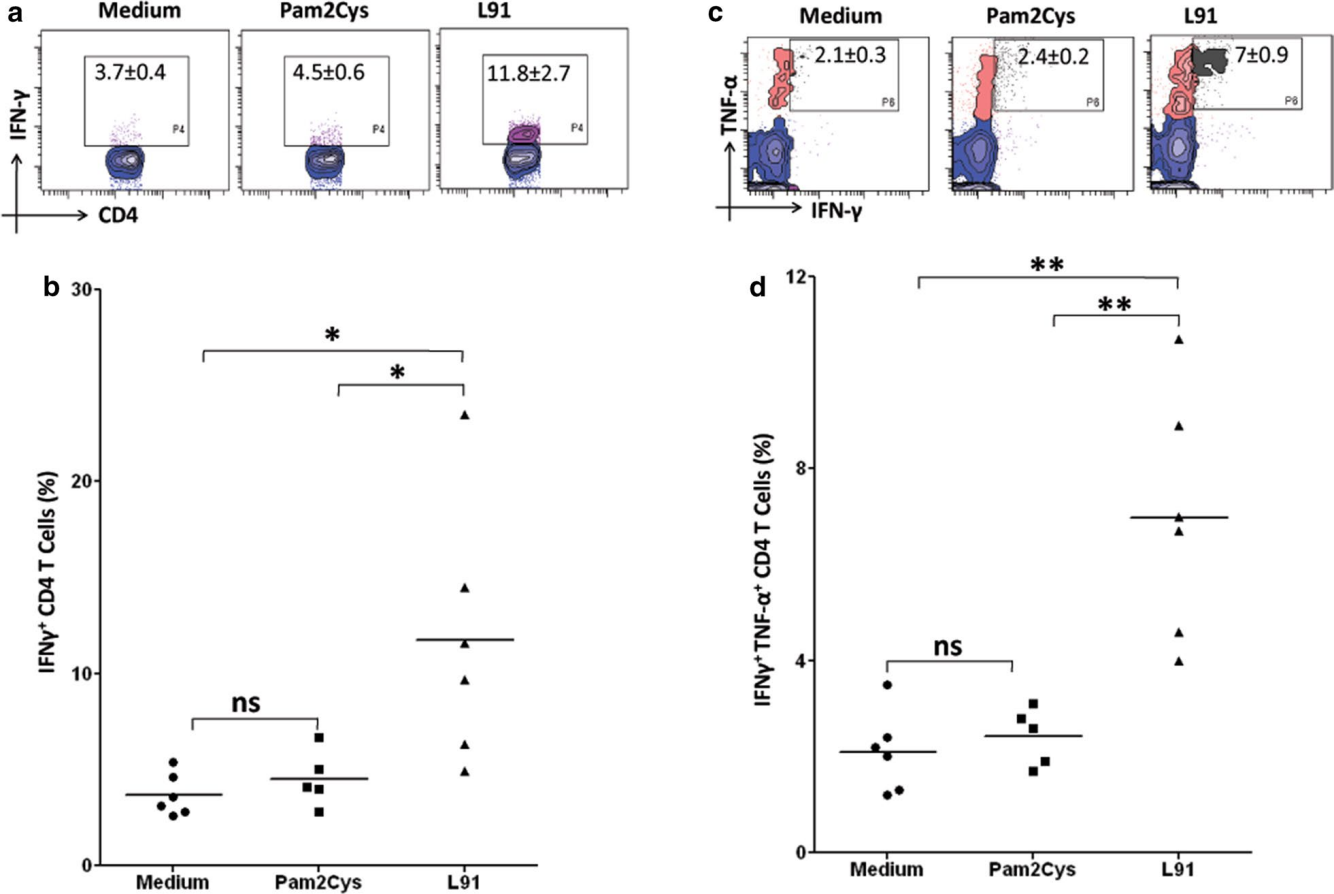
L91 elicits polyfunctional Th1 cells in BCG vaccinated human subjects of the non-TB endemic population. Human PBMCs obtained from BCG-vaccinated individuals from non-TB endemic population were analyzed by flow cytometry for the expression of **a** IFN-γ and **b** represented by dot plots and (**c**, **d**) IFN-γ and TNF-α together. Each dot corresponds to one human subject. Data are shown as Mean ± SEM. *p ≤ 0.05, **p ≤ 0.005, *ns* not significant

**Fig. 8:**
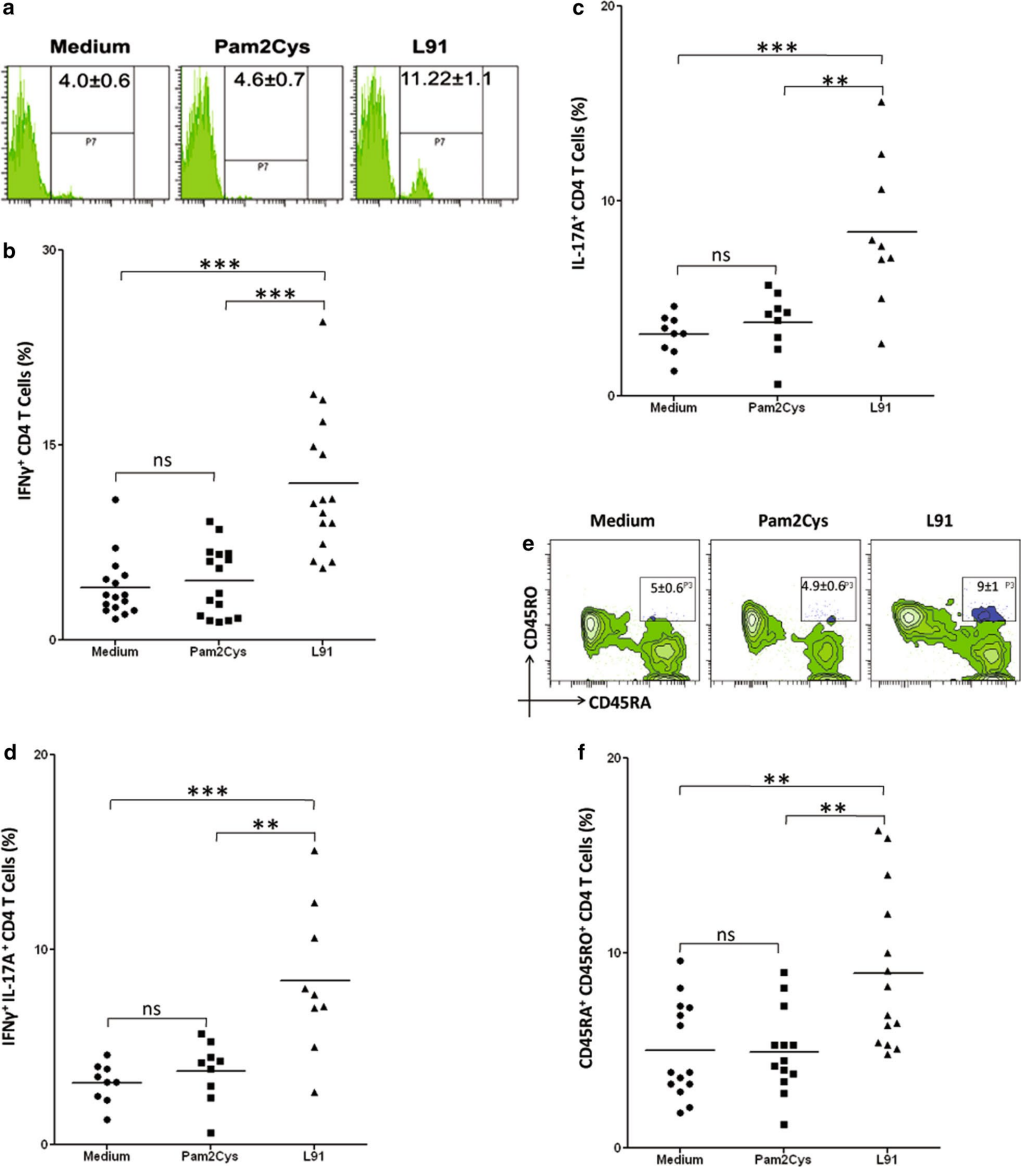
L91 elicits long lasting Th1 and Th17 immune response of BCG vaccinated volunteers. PBMCs of BCG vaccinated human subjects were stimulated with L91 and control cultures with Pam2Cys and medium for 96 h and analyzed for the expression of memory markers and cytokines by flow cytometer. **a** Histogram and **b** scatter plot depicts percent population CD4 T cell expressing IFN-γ (n = 16). Likewise, **c** scatter dot plot represents percent population of CD4 T cell expressing IL-17A (n = 9). **d** Polyfunctional T cells were identified on CD4 gated T cells co-expressing IFN-γ and IL-17A (n = 9). **e** Histogram and **f** scatter dot plot represent the percentage of CD45RA^+^CD45RO^+^ memory CD4 T cells in BCG immunized human subjects (n = 14). Data are shown as Mean ± SEM. **p ≤ 0.005, ***p ≤ 0.0005, *ns* not significant

We next observed that L91 stimulation improved the generation of Th1 cells, as it significantly (*p* ≤ 0.05) expanded the percentage of IFN-γ^+^ CD4 T cells. Further, we observed increased frequency (*p* ≤ 0.005) of polyfunctional Th1 cells expressing both IFN-γ and TNF-α (Fig. 7a–d). The results were obtained using blood from the non-TB endemic Australian population. Furthermore, we also monitored the efficacy of L91 using PBMCs from BCG-vaccinated healthy volunteers of the TB-endemic Indian population. It is worth to mention here that the BCG has failed to protect Indian population from *Mtb* [4]. We observed a significant augmentation in Th1 and Th17 immunity, as documented by the increased percentage of IFN-γ^+^ (*p* ≤ 0.0005) and IL-17A^+^ (*p* ≤ 0.005) CD4 T cells, respectively (Fig. 8a–c). Further, enhanced frequency (*p* ≤ 0.005) of IFN-γ^+^ and IL-17A^+^ polyfunctional Th17 cells was detected (Fig. 8d). Furthermore, expansion (*p* ≤ 0.005) of the pool of the memory precursors of CD4 T cells (CD45RA^+^CD45RO^+^) was noted (Fig. 8e). These results suggest that L91 efficiently activated polyfunctional human Th1 cells and Th17 cells and expanded the pool of memory CD4 T cells. Thus, has a great potential to reinvigorate the efficacy of BCG vaccine in TB endemic and non-endemic population.

## Discussion

The slow progress of the ‘Stop TB Program’ and the emergence of drug resistant strains of *Mtb* poses an urgent challenge for the scientific community to develop an effective vaccine against *Mtb*. BCG is the only currently available vaccine, which is widely administered worldwide [10]. Nevertheless, TB accounts for 9.6 million new cases and 1.5 million mortality annually [1]. In many ways BCG is a controversial vaccine, since it protects 80% of individuals in non-TB endemic regions and 0% in TB-endemic zones [7, 29]. BCG protects children but not adults living in TB-endemic regions, further demonstrating its variable efficacy [5, 10, 30]. Many approaches to improve BCG vaccine have been tried, which includes recombinant BCG with different antigenic formulations [29–31]. However, the introduction of a globally effective vaccine candidate, at least in TB-endemic zones, is yet to be achieved. The high protective efficacy of BCG in the developed world and children of TB-endemic sectors suggest that BCG has the adequate antigenic repertoire to protect against *Mtb* [23]. Nevertheless, BCG efficacy wanes with age, which indicates its failure to elicit life-long immunity [32]. In this context, several approaches are being tried to boost BCG ability to generate enduring memory T cells and protection against TB [8, 9].

L91 is a chimeric peptide comprising of MHC-II binding peptide and Pam2Cys and promotes the generation of long-term memory CD4 T cells [10]. Therefore, we were encouraged to test the potential of L91 to bolster BCG efficacy by promoting the persistence of memory CD4 T cells and consequently long lasting protection against *Mtb*.

We carried out a study in which L91 was used to boost the immune response primed by BCG vaccination. As compared to BCG group, the following major findings have been obtained: (i) significant reduction in the number of CFU in the lungs and diminished pathological changes in the *Mtb* infected mice; (ii) higher proliferation of the CD4 T cells and upregulated expression of IFN-γ and IL-17A; (iii) robust increase in the pool of multifunctional Th1 cells (IFN-γ^+^/TNF-α^+^) and Th17 cells (IL-17^+^/IFN-γ^+^/CXCR3^+^/CCR6^+^); (iv) expansion in the percentage of central and effector memory CD4 T cells; (v) the mechanism involved in the reduction of bacterial burden was through iNOS and TNF-α; (vi) the PBMCs obtained from the BCG vaccinated volunteers showed increase in the frequency of polyfunctional Th1 cells, Th17 cells and memory CD4 T cells on in vitro exposure to L91.

One of the main reasons associated with the weak efficacy of BCG vaccine to protect against *Mtb* is attributed to the generation of Tregs following immunization, which dampens Th1 immunity through release of IL-10 [33–35]. The decrease in the frequency of Tregs in the BCG-L91 group compared to BCG group clearly indicates the importance of L91 boosting in reducing the number of Tregs. The inhibition in the development of Tregs by BCG-L91 may be due to the augmented secretion of IFN-γ by Th1 cells [36, 37]. Furthermore, the signaling events stimulated by TGF-β are negatively regulated by IFN-γ [38]. TGF-β is responsible for the differentiation of naïve CD4 T cells to Tregs. IFN-γ-mediated phosphorylation of STAT1 leads to the expression of T-bet and Smad7 [38, 39], which is known to suppress the regulatory function of Tregs [40].

It has also been well documented that *Mtb* drives Th1 cells to exhaustion [11]. Th1 cells play a crucial role in protecting against *Mtb* [10, 11]. Recently, we have demonstrated that triggering of TLR-2 with Pam2Cys can enhance the generation of memory Th1 cells and rescues them from exhaustion by downregulating PD-1 and Tim-3 and amplifying co-stimulatory signals and the secretion of pro-inflammatory cytokines [10, 11]. TLR-2 agonist Pam2Cys is a major component of L91. TLR-2 signaling not only rescues T cells from exhaustion but also stimulates both innate and adaptive immunity. Further, the higher susceptibility of TLR-2^−/−^ animals to *Mtb* indicates an important role of TLR-2 in protection [41, 42].

The induction of Th1 immunity plays a fundamental role in protecting against intracellular pathogens such as *Mtb* [7, 8, 10]. Further, MyD88^−/−^ animals with partially compromised Th1 immunity are more susceptible to TB [15]. We demonstrated that the L91 booster substantially augmented Th1 immunity, as evidenced by improvement in IFN-γ secretion. Pam2Cys is known to stimulate DCs to release IL-12 through TLR-2 signaling [10]. IL-12 is a differentiating factor for Th1 cells. Thus, L91 preferentially expands Th1 cells. Furthermore, we noted the generation of Th17 cells in the animals vaccinated with BCG-L91. Recently, it was demonstrated that Th17 cells confer protection against *Mtb* by recruiting Th1 cells to the site of infection. Th17 cells show a robust effect on chemokine-mediated infiltration of macrophages and neutrophils at the site of infection [17, 18].

It has been shown that the protective role of polyfunctional Th1 cells (IFN-γ^+^/TNF-α^+^) and Th17 cells (IFN-γ^+^/IL-17A^+^) is qualitatively superior to their single cytokine secreting counterparts, since they can effectively restrict the survival of *Mtb* [10, 17]. Interestingly, we found that BCG-L91 immunization expanded IFN-γ and TNF-α secreting polyfunctional Th1 cells. IFN-γ is known to activate macrophages and increase their bactericidal effect and TNF-α restricts the growth of *Mtb* [43, 44]. Such Th17 cells are reported to have a better protective capacity than single cytokine secreting cells [17, 18, 45]. Further, Th17 cells expressing CXCR3^+^CCR6^+^ are associated with protection against *Mtb*, while those displaying CCR6^+^ CCR4^+^ are involved in autoimmunity [17, 45]. We found in this study that L91 preferentially expanded polyfunctional Th17 cells displaying CXCR3 and CCR6. Consequently, suggesting non-pathogenic nature of Th17 cells.

The importance of NO has been well documented in the killing of *Mtb* by inducing apoptosis of infected cells [27]. L91 activates DCs and augments the expression of iNOS and TNF-α. Both NO and TNF-α cumulatively protect the host from *Mtb* and the mechanism deciphered is by inducing apoptosis of *Mtb*-infected cells. Apoptosis releases intracellular bacteria, which provides an opportunity for the activated macrophages to engulf and eliminate them [27]. We have shown that a booster dose of L91 efficiently bolsters the protective efficacy of BCG and significantly constrains bacterial burden in the lungs and spleen even after a lengthy period of 229 days of vaccination. The results of protection study suggest that L91 has a unique capability of generation and maintenance of long-lasting memory CD4 T cells and protection against *Mtb* [10].

Finally, we validated the efficacy of L91 employing PBMCs of BCG vaccinated healthy adult volunteers from TB-endemic and non-endemic zones. Importantly, CD4 T cells exhibited an enhacement in the expression of IFN-γ and IL-17A on in vitro stimulation with L91. Furthermore, an increase in the frequency of polyfunctional Th1 cells and Th17 cells was detected. Finally, it is worth to mention here that a remarkable expansion in the pool of memory CD4 T cells was found in our study, which illustrates the role of L91 in reinvigorating BCG efficacy to evoke long-lasting immunity responsible for protection against *Mtb*.

## Conclusions

This study indicates that BCG priming followed by L91 boosting could efficiently overcome the low long-term protective efficacy associated with BCG vaccination by generating enduring memory T cells and protection against *Mtb*. The possible mechanism involved is through the involvement of both innate (NO and TNF-α) and adaptive (long-lasting polyfunctional Th1 cells and Th17 cells) immunity. In future, this vaccination strategy of BCG-L91 vaccine may be developed into an effective strategy to control TB in the TB-endemic population.

## Additional files

**Additional file 1: Figure S1.** *Gating strategy for monitoring the expression of FoxP3, PD*-*1, Tim*-*3, IFN*-*γ, IFN*-*γ* ^+^ *TNF*-*α, IL*-*17A, IL*-*17A* ^+^ *IFN*-*γ, CD62L* ^+^ *CD44 and CD127 on CD4* ^+^ *T cells*. CD4^+^ T cells were stained with the fluorochrome labeled Abs to FoxP3, PD-1, Tim-3, IFN-γ, IFN-γ^+^TNF-α, IL-17A, IL-17A^+^IFN-γ, CD62L^+^CD44, CD127. The P1 gate was made on lymphocyte zone and P2 gate on SSC-A and CD4^+^ T cells. The expression of FoxP3, PD-1, Tim-3, IFN-γ, IFN-γ^+^TNF-α, IL-17A, IL-17A^+^IFN-γ, CD62L^+^CD44 and CD127 was observed on P2 gated population (CD4^+^ and SSC-A^+^). The unstained cells failed to show any CD4^+^ T cell population.

**Additional file 2: Figure S2.** *Gating procedure for monitoring the expression of CXCR3* ^+^ *CCR6 on IFN*-*γ* ^+^ *IL*-*17A expressing CD4 T cells*. The P1 gate was made on lymphocyte zone and P2 gate on SSC-A and CD4^+^ T cells. The display of IL-17A^+^IFN-γ (P3 gate) was monitored on P2 zone (CD4^+^ and SSC^+^). The expression of CXCR3^+^CCR6 was examined on P3 region (IL-17A^+^IFN-γ positive cells). The unstained cells failed to show any CD4^+^ T cell population.

## Abbreviations

BCG: Bacillus Calmette–Guérin
*Mtb*: *Mycobacterium tuberculosis*
CD: cluster of differentiation
IFN: interferon
TNF: tumor necrosis factor
IL: interleukin
PD: programmed cell death
PBMC: peripheral blood mononuclear cell
TB: tuberculosis
TLR: toll like receptor
Acr: alpha-crystallin
PBS: phosphate buffered saline
CFU: colony forming unit
ST-CF: short term culture filtrate
CFSE: carboxyfluoresceinsuccinimidyl ester
PMA: phorbol myristate acetate
CCR/CXCR: chemokine and cytokine receptor
GM-CSF: granulocyte–macrophage colony-stimulating factor
LPS: lipopolysaccharides
RIPA: radioimmunoprecipitation assay buffer
iNOS: nitric oxide synthases
MHC: major histocompatibility complex
NO: nitric oxide
SNs: supernatants
