# Supplementary figures and images for: A lipidated peptide of Mycobacterium tuberculosis resuscitates the protective efficacy of BCG vaccine by evoking memory T cell immunity
Source: J Transl Med. 2017 Oct 6;15:201. doi: 10.1186/s12967-017-1301-x (PMC6389088; doi:10.1186/s12967-017-1301-x)

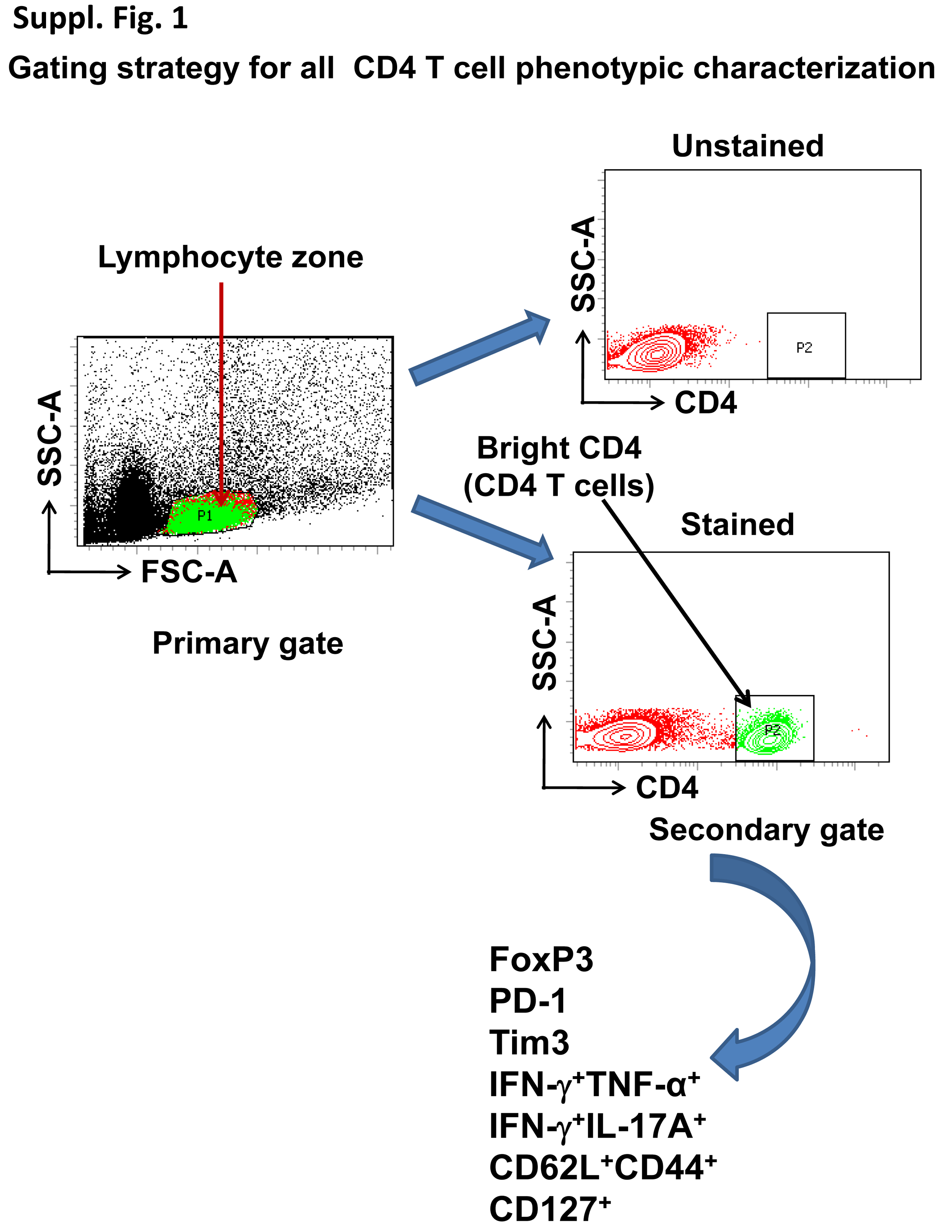

Supplement: Supplementary file 1 — Additional file 1: Figure S1. Gating strategy for monitoring the expression of FoxP3, PD-1, Tim-3, IFN-γ, IFN-γ + TNF-α, IL-17A, IL-17A + IFN-γ, CD62L + CD44 and CD127 on CD4 + T cells. CD4+ T cells were stained with the fluorochrome labeled Abs to FoxP3, PD-1, Tim-3, IFN-γ, IFN-γ+TNF-α, IL-17A, IL-17A+IFN-γ, CD62L+CD44, CD127. The P1 gate was made on lymphocyte zone and P2 gate on SSC-A and CD4+ T cells. The expression of FoxP3, PD-1, Tim-3, IFN-γ, IFN-γ+TNF-α, IL-17A, IL-17A+IFN-γ, CD62L+CD44 and CD127 was observed on P2 gated population (CD4+ and SSC-A+). The unstained cells failed to show any CD4+ T cell population. [file 12967_2017_1301_MOESM1_ESM.tif]

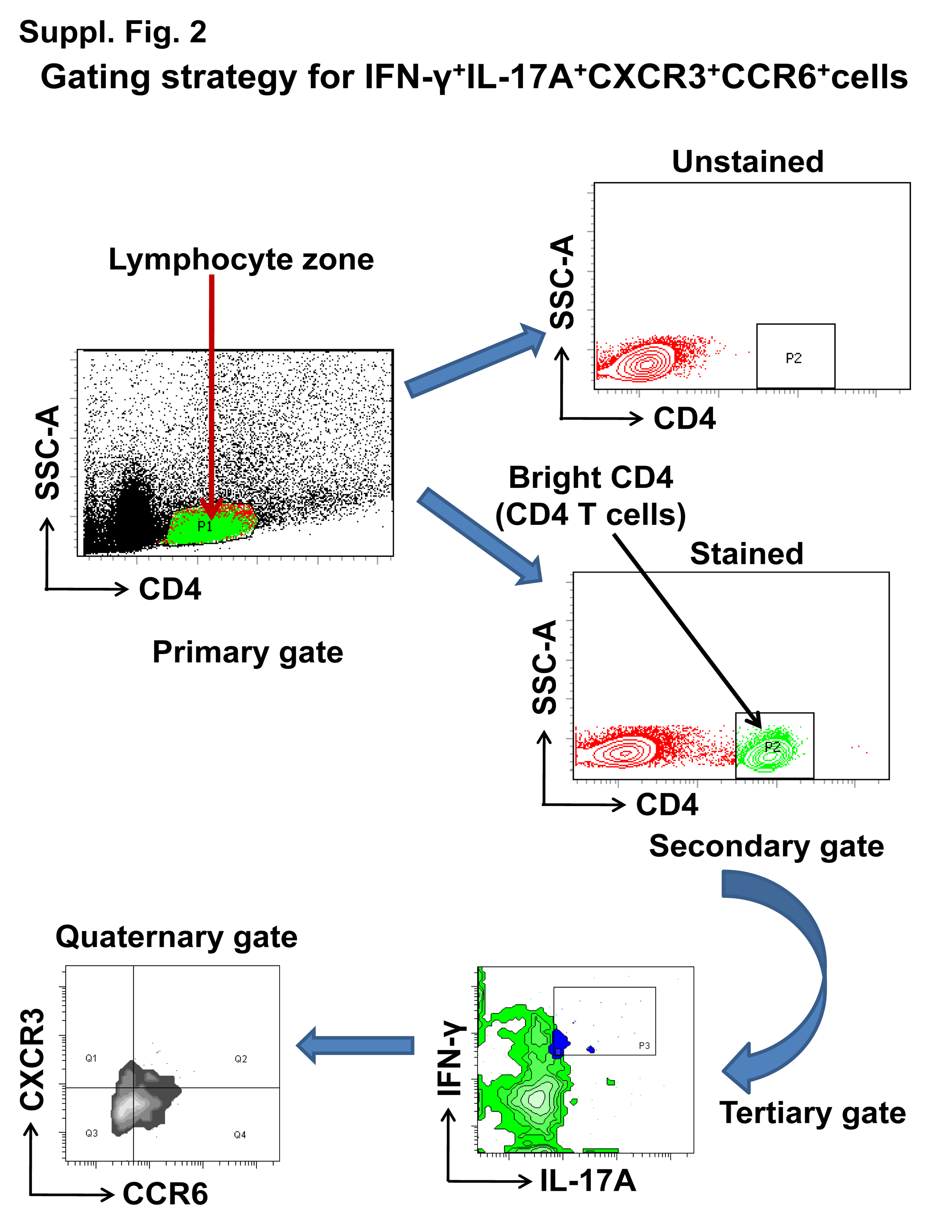

Supplement: Supplementary file 2 — Additional file 2: Figure S2. Gating procedure for monitoring the expression of CXCR3 + CCR6 on IFN-γ + IL-17A expressing CD4 T cells. The P1 gate was made on lymphocyte zone and P2 gate on SSC-A and CD4+ T cells. The display of IL-17A+IFN-γ (P3 gate) was monitored on P2 zone (CD4+ and SSC+). The expression of CXCR3+CCR6 was examined on P3 region (IL-17A+IFN-γ positive cells). The unstained cells failed to show any CD4+ T cell population. [file 12967_2017_1301_MOESM2_ESM.tif]
